# COVID-19 Vaccination Behavior Among Frontline Healthcare Workers in Pakistan: The Theory of Planned Behavior, Perceived Susceptibility, and Anticipated Regret

**DOI:** 10.3389/fpsyg.2022.808338

**Published:** 2022-04-14

**Authors:** Muhammad Khayyam, Shuai Chuanmin, Muhammad Asad Salim, Arjumand Nizami, Jawad Ali, Hussain Ali, Nawab Khan, Muhammad Ihtisham, Raheel Anjum

**Affiliations:** ^1^School of Economics and Management, China University of Geosciences (Wuhan), Wuhan, China; ^2^HELVETAS Swiss Intercooperation, Islamabad, Pakistan; ^3^College of Management, Sichuan Agricultural University Chengdu Campus, Wenjiang, China; ^4^College of Landscape Architecture, Sichuan Agricultural University, Wenjiang, China; ^5^Department of Economics, Abdul Wali Khan University, Mardan, Pakistan

**Keywords:** COVID-19 vaccination, SARS-CoV-2, frontline healthcare workers, anticipated regret, perceived susceptibility, the theory of planned behavior

## Abstract

Healthcare workers in Pakistan are still fighting at the frontline to control the spread of severe acute respiratory syndrome coronavirus-2 (SARS-CoV-2) and have been identified as the earliest beneficiaries for COVID-19 vaccination by the health authorities of the country. Besides, the high vaccination rates of frontline healthcare workers (FHWs) are essential to overcome the ongoing pandemic and reduce the vaccines hesitancy among the general population. The current research employed the theory of planned behavior (TPB) to investigate the COVID-19 vaccination behavior among FHWs in Pakistan as well as the predictors of such behavior. Following the epidemic control and prevention policies, a sample of 680 FHWs were accessed to fill in the questionnaire evaluating the components of the TPB. Moreover, the potential role of anticipated regret (AR) and perceived susceptibility (PS) on COVID-19 vaccination behavior was also assessed. The partial least square structural equation modeling (PLS-SEM) results revealed that the TPB components, as well as the AR, have positive associations with the COVID-19 vaccination behavior. The results further confirmed that PS positively affects the anticipated regret, attitude (ATT), and subjective norm (SN) to vaccinate against SARS-CoV-2. The perceived susceptibility also has a positive association with COVID-19 vaccination behavior through the mediation of anticipated regret, ATT, and SN. Our findings highlighted the importance of COVID-19 vaccination among healthcare workers, which can be applied to reduce vaccine hesitancy among the general public.

## Introduction

The novel coronavirus disease 2019 is a global pandemic caused by severe acute respiratory syndrome coronavirus-2 (SARS-CoV-2). Globally, as of 4:59pm CEST, 1 April 2022, there have been 486,761,597 confirmed cases of COVID-19, including 6,142,735 deaths, reported to WHO (COVID-19 Dashboard, 2020). In response, the WHO led the global efforts in fighting against the pandemic through preventing, diagnosing, and treating this exclusive pathogen. To restore normal civilian life and economic rehabilitation, the development of a vaccine is the most promising mean. For this reason, a simultaneous and sustained race to discover a safe and effective vaccine was initiated in the first half of 2020 by more than 90 vaccine development companies worldwide. Sooner than expected, on 31st December 2020, a vaccine to COVID-19 developed by Pfizer-BioNTech was approved by the United States Food and Drug Administration (FDA), which was quickly followed by authorizations to other vaccines, namely, Moderna, AstraZeneca, and Janssen (GAVI The Vaccine Alliance, 2021). Till now, more than 200 COVID-19 vaccines are under development, of which more than 106 vaccines have entered the clinical trials, whereas, 21 vaccines have been rolled out worldwide (GAVI The Vaccine Alliance, 2021).

Pakistan till the first quarter of 2022, has recorded the 26th highest number of confirmed COVID-19 cases (1,480,592) and death toll (29,731; COVID-19 Situation, 2022). The country is facing a huge economic disruption with an unknown future (Ashfaq and Bashir, 2021). A vaccine to mitigate the spread of coronavirus is perhaps the only hope to control the situation. Following the National Command and Operation Center (NCOC) of Pakistan’s approval of different kinds of vaccines, the country has started a phased vaccine roll out in March 2021. Subsequent to the guidelines of NCOC, the frontline healthcare workers (FHWs) and elder citizens were scheduled to be the first beneficiaries of the vaccine followed by other age groups of the population. Till the first quarter of 2022, a total of 194,492,475 doses of different kinds of COVID-19 vaccines have been administered in Pakistan. Out of total administered doses 115,238,268 (52.16%) population is partially vaccinated, whereas, 89,853,639 (40.67%) population is fully vaccinated. Due to the advent of COVID-19 variants, 3,361,160 (1.52%) of the population is also vaccinated with booster doses. However, the excessive efforts for COVID-19 vaccines’ availability cannot promise the end of the pandemic due to the ongoing vaccine hesitancy and anti-vaccine campaigns worldwide (Shih et al., 2021).

The healthcare workers in Pakistan are fighting in the frontline against the ongoing pandemic. Due to the constant exposure to the COVID-19-infected patients, these FHWs are at high risk of infection. Based on the NCOC estimates, at least 40% of healthcare workers have been diagnosed with SARS-CoV-2, and thus, their vaccination against the disease is deemed important. The literature regarding the acceptability of COVID-19 vaccines among healthcare workers is yet limited. However, most of the investigations revealed controversial results (Shih et al., 2021). There are ample cases available showing that healthcare workers themselves were vaccine-hesitant that also affected its acceptance in the general public (Grech et al., 2020; Wong et al., 2020; Xiao and Wong, 2020; Kumar et al., 2021). Studies conducted in Greece (Zampetakis and Melas, 2021) and the Democratic Republic of the Congo (Nzaji et al., 2020) revealed that only a meager sum of healthcare workers (16 and 0.2%, respectively) were willing to be immunized against COVID-19. Similarly, the survey reports in China also reported the unwillingness of Chinese nurses to get COVID-19 vaccination (Li et al., 2021). In contrast, the healthcare workers in Belgium, France, and Canada reported a comparatively high percentage of willingness to get COVID-19 vaccination (Verger et al., 2021). There could be several factors to underlie this behavior including low perceived risk of infection (Harapan et al., 2020), low perceived benefits (Wong et al., 2020), fear of side effects (Fakonti et al., 2021), and concerns regarding its safety (Wang et al., 2021) and efficacy (Grech et al., 2020).

In Pakistan, none of the researchers have explored the healthcare workers’ behavior on vaccination against new coronavirus SARS-CoV-2. Given the enormous role of inoculated healthcare workers in shaping the general population’s willingness to vaccinate against the disease, and as the availability of a vaccine does not essentially translate into its adoption, the current research thus aims to investigate the actual behavior of healthcare workers in Pakistan to vaccinate against SARS-CoV-2. The review of relevant literature suggests that the theory of planned behavior (TPB) and health belief model (HBM) are the widely adopted socio-psychological frameworks to predict individuals’ health behavior (Khayyam et al., 2021). The current research applies the theoretical framework of the TPB to examine the COVID-19 vaccination behavior of FHWs in Pakistan. In addition to the TPB, other potential factors, such as perceived susceptibility (PS) of HBM and anticipated regret (AR), were also added in the classical TPB framework given that these factors can influence individuals’ behavioral intentions, particularly when it comes to health-related concerns.

During the last decades, the individuals’ vaccination uptake behavior has been thoroughly examined. Numerous socio-psychological theories which act as the foundation to examine the intended behavior have been acknowledged by literature. The TPB is an expectancy-value framework that has been widely applied to predict several health-related behaviors (Wolff et al., 2011), that includes intentions to undergo genetic screening, intentions to uptake Human Papillomavirus Vaccine (HPV; Li and Li, 2020), intentions to uptake influenza vaccine (Alhalaseh et al., 2020), and even intentions to uptake COVID-19 vaccination (Alhalaseh et al., 2020; Shmueli, 2021; Wolff, 2021). The TPB suggests that precise behavior is assessed through the behavioral intentions to perform it (Ajzen, 2012). The intention itself is further influenced by other components known as attitude (ATT), subjective norms (SN), and perceived behavioral control (PBC; Yzer, 2017). AR is the experience or feelings of regret for a current situation that we think we might feel in the future, typically about decisions we currently consider making. Anticipated regret in current research is the feelings of a future regret a healthcare worker will feel for refusing the COVID-19 vaccine and subsequently contracting the virus. Whereas, PS is an individual perception of being vulnerable to contracting a disease. In the present research, the perceived susceptibility is the extent that frontline health workers would believe themselves at high risk of being infected with COVID-19 if they are not immunized against the disease.

### Aims and Hypothesis

Subsequent to the TPB, the first aim of the current study is to explain the vaccination behavior of the FHWs in Pakistan to vaccinate against the new coronavirus SARS-CoV-2. A second aim is to investigate how anticipated regret plays its role in the current decision of FHWs to uptake COVID-19 vaccines. Based on the prime reason of being more vulnerable to SARS-CoV-2 and thus avoiding possible future infections, the third aim of the study is to investigate the role of perceived susceptibility as a background variable shaping the COVID-19 vaccination behavior among FHWs in Pakistan.

Attitude is the psychological propensity that describes how an individual evaluates self-performance (favorable or unfavorable), which predicts the intention, and consequently the actual behavior (Khayyam et al., 2021). Consequent to the TPB, when an individual holds positive attitudes toward a certain behavior, their willingness to engage in that behavior is relatively high (Yzer, 2017). Similarly, the positive associations between attitude and intentions to vaccinate against a specific disease have been highlighted by several studies. For instance, Li and Li (2020) argued that young Chinese women who have a positive attitude toward HPV vaccination would be more willing to uptake the HPV vaccine than those who hold negative attitudes. Similarly, positive associations between attitude and intentions to uptake the COVID-19 vaccination have been reported in several studies (e.g., Cai et al., 2021; Fakonti et al., 2021; Wolff, 2021). Therefore, in our study, we aimed that the healthcare workers in Pakistan that are fighting at the frontline to combat the spread of the virus, while treating the infected individuals would be more willing to immunize against the SARS-CoV-2.

Subjective norm, the second most pertinent determinant of intention also plays an important role to perform the behavior of interest (Khayyam et al., 2021). It refers to the perceived external pressure from important others to perform or not to perform a specific behavior (Yzer, 2017). Based on TPB, individuals who consider the certain behaviors of their important referent (such as family and friends) as important and imperative, are more inclined to perform that behavior (Park, 2000). Kok et al. (2011) explored that the healthcare workers in Netherland feel supported by their peers and department heads when planning to decide about the influenza vaccination. Pakistani society is formed by collectivistic cultural, social, and religious identity where group affiliation is considered important, therefore, the subjective norm to play a vigorous role in influencing the behavioral intentions to uptake COVID-19 vaccines is quite reasonable.

Following the TPB, PBC is the perceived easiness or ability to execute the behavior of interest (Yzer, 2017). The PBC predicts both intentions and actual behavior (Conner and Armitage, 1998); however, the strength of the relationship concerning the intentions and actual belief varies across studies. Ajzen (2002) argued that the individuals who feel confident of being able to execute the behavior are more likely to accomplish it. In the current study, those frontline health workers who perceive themselves as being able to uptake COVID-19 vaccination are more likely to get vaccinated against SARS-CoV-2. Thus, we hypothesize as follows:

*H1:* Attitudes predict the COVID-19 vaccination behavior of FHWs to vaccinate against SARS-CoV-2.

*H2:* Subjective norms predict the COVID-19 vaccination behavior of FHWs to vaccinate against SARS-CoV-2.

*H3:* Perceived behavioral control predicts the COVID-19 vaccination behavior of FHWs to vaccinate against SARS-CoV-2.

**Figure 1:**
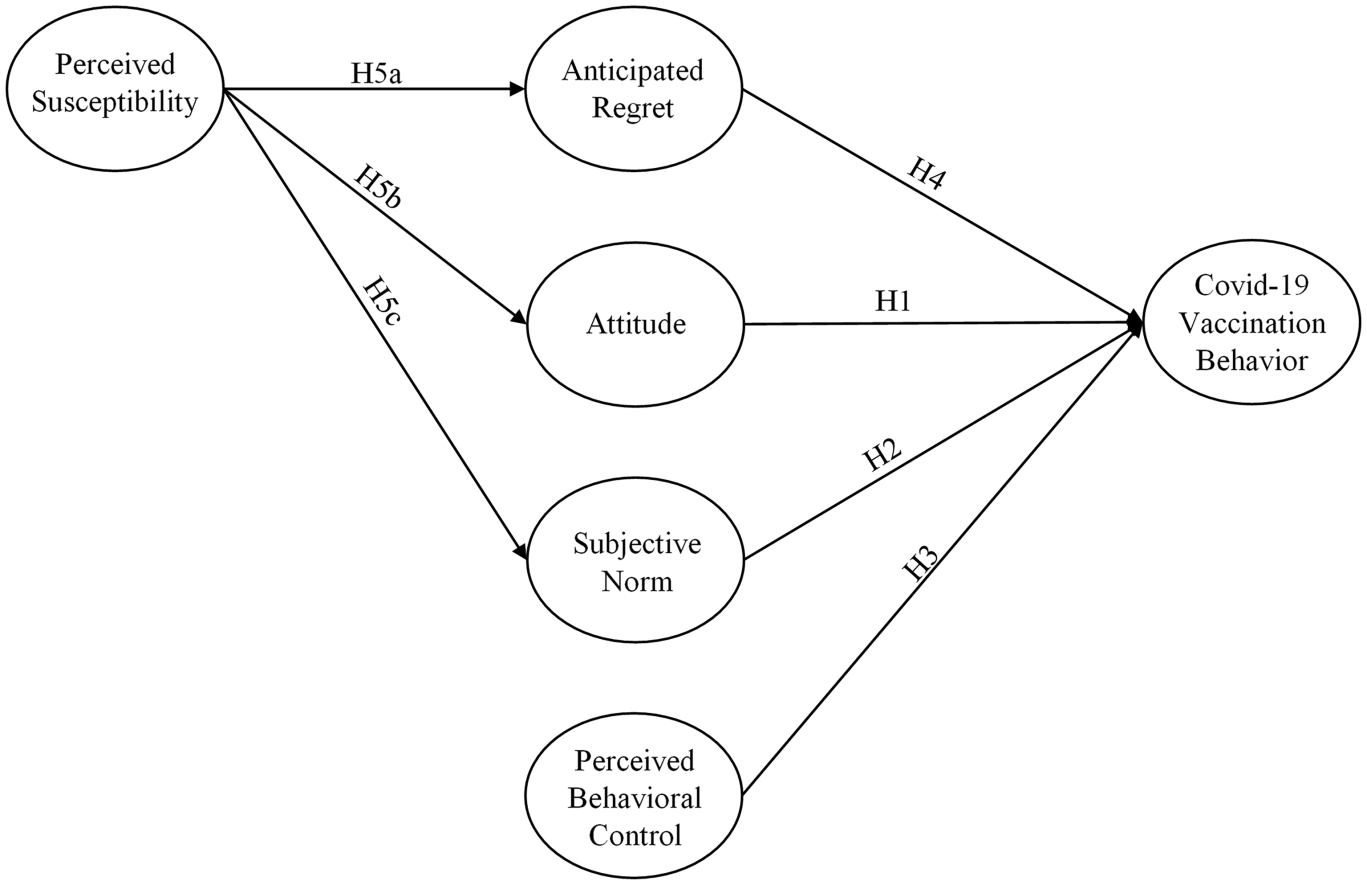
Hypothesized COVID-19 vaccination model.

The TPB, as suggested by academic literature, allows several background variables (perceived severity, risk perception, optimistic bias, and interpersonal discussion). These variables can serve as important factors to affect an individual’s belief regarding health issues. The integrated TPB framework developed for this study (Figure 1) contemplated anticipated regret as an additional component of TPB and perceived susceptibility as a background variable to better predict the COVID-19 vaccination behavior of FHWs. In fact, anticipated regret and perceived susceptibility are the components whose nature and importance in health-related issues are more relevant to the TPB constructs (Christy et al., 2016; Penţa et al., 2020; Wong et al., 2020; Wolff, 2021).

Brewer et al. (2016) defined regret as, “an aversive cognitive emotion that we experience when realizing or imagining that our current situation would have been better, if only we had decided differently,” whereas, anticipated regret is the feelings of regret in a current situation that we think we might feel in the future (Penţa et al., 2020). According to regret theory, individuals assume the feelings they might experience in the future regarding current decisions (Wolff, 2021). Brewer et al. (2016) conducted a meta-analysis to see if anticipated regret influences a variety of health-related decisions, including vaccination. Furthermore, their analysis showed a lower rating of anticipated regret for vaccination than anticipated regret for refusing vaccination. This could be attributed to the reason that individuals anticipate less regret for easily admissible decisions than for less admissible ones. In contrast, the recent studies of Caso et al. (2021) and Galanis et al. (2021) reported a higher level of anticipated regret for vaccination among a specific groups who abide to the so-called “no-vax” groups. Several studies (e.g., Christy et al., 2016; Ng et al., 2020; Penţa et al., 2020) investigated the potential role of anticipated regret to uptake the HPV and influenza vaccinations respectively. In this research, we anticipate that the FHWs in Pakistan have less regret and self-blame to uptake COVID-19 vaccination. Therefore, we hypothesize as follows:

*H4:* Anticipated regret predicts the COVID-19 vaccination behavior of FHWs to vaccinate against SARS-CoV-2.

Perceived susceptibility, refers to the belief that there is an absolute risk of being infected with a disease (Zampetakis and Melas, 2021). The perceived risk to vaccine-preventable disease may also influence the gain-vs.-loss decisions. For instance, Li and Li (2020) identified perceived susceptibility as one of the important factors affecting the behavioral intentions of young Chinese women to uptake HPV vaccination. Similarly, Alhalaseh et al. (2020), Ng et al. (2020), and Tao et al. (2021) also explored that perceived susceptibility plays a vital role to shape individuals’ intentions to uptake influenza vaccine in Jordan, China, and Hong Kong, respectively. Moreover, considering the seriousness of SARS-CoV-2, several recent research studies (e.g., Wong et al., 2020; Huynh et al., 2021; Iacob et al., 2021; Tao et al., 2021) also explored that the perceived susceptibility associated with SARS-CoV-2 (i.e., high infectivity ratio) contributes to higher intentions of COVID-19 vaccination among health workers and the general population as well.

Perceived susceptibility in the current research is the extent that FHWs in Pakistan believe themselves at a higher risk to contract the deadly virus if they would not uptake the COVID-19 vaccines. Furthermore, according to HBM, perceived susceptibility is an important factor to predict human behavior (Wong et al., 2020). However, based on the extensive literature review, very scant researches have examined the associations between perceived susceptibility, attitude, and subjective norms. More precisely, in the context of the current research, none of the studies have investigated the extent to which the relationship between perceived susceptibility and COVID-19 vaccination behavior is mediated by anticipated regret, attitude, and subjective norm. Figure 1 summarizes the proposed mediated relationships among the variables.

Researchers including Yang (2015) and Shmueli (2021) argued that attitudes and subjective norms might influence the health behavioral change with the existence of some cues to action. In particular, when an external stimuli exists in the social/external environment that cue to take the recommended actions will more likely effect the attitudes and motivation to comply with important referents. The present study firstly assumed perceived susceptibility as the external stimuli that influences the FHWs attitudes and motivations to comply with important referents to vaccinate against SARS-CoV-2. Secondly, the study also assumed that the considered external stimuli influences the anticipated regret of FHWs.

Considering the severity of SARS-CoV-2, we believe that the healthcare workers in Pakistan who are fighting at the frontline to combat the virus with 40% of workers already been infected will have a lower anticipated regret to vaccinate against the disease than those who are not vaccinating. In addition, we assume that the more FHWs in Pakistan believed themselves of being vulnerable to SARS-CoV-2, the more they will be holding a positive attitude to uptake COVID-19 vaccination. Similarly, we contend that the positive relationship between perceived susceptibility and subjective norm means FHWs will comply with the guidelines and protocol provided by NCOC. Thus, we hypothesize the following hypothesis:

*H5a:* Perceived susceptibility has a negative effect on the anticipated regret of FHWs to vaccinate against SARS-CoV-2.

*H5b:* Perceived susceptibility has a substantial positive effect on the attitude of FHWs to vaccinate against SARS-CoV-2.

*H5c:* Perceived susceptibility has a substantial positive effect on the subjective norm of FHWs to vaccinate against SARS-CoV-2.

Finally, following the conceptual formwork developed by Yang and other it is also proposed in the current research model, that the anticipated regret, attitude, and subjective norm will mediate the relationships between perceived susceptibility (as an external stimuli) and the COVID-19 vaccination behavior (health behavioral change) of FHWs in Pakistan. Based on the extensive review of available literature in the subject matter, very scant researchers have analyzed that the relationship between perceived susceptibility and COVID-19 vaccination behavior is mediated by the mentioned variables (anticipated regret, attitude, and subjective norm). Thus, the following hypotheses were posed:

*H6a:* Perceived susceptibility has a potential positive and indirect association with COVID-19 vaccination behavior of FHWs, through anticipated regret.

*H6b:* Perceived susceptibility has a potential positive and indirect association with COVID-19 vaccination behavior of FHWs, through attitude.

*H6c:* Perceived susceptibility has a potential positive and indirect association with COVID-19 vaccination behavior of FHWs, through the subjective norm.

## Materials and Methods

### Procedures and Participants

The proposed model was examined using a quantitative technique in the current study. The information was gathered using well-organized self-administered questionnaires that were physically distributed among Pakistani FHWs in the month of July and August 2021. The targeted population in the current research were those including doctors, health technicians, nurses, midwives, and community health workers in two cities (Peshawar and Mardan) of Khyber Pakhtunkhwa (KP) province, Pakistan. All of the responders were frontline workers who were at a greater risk of catching the new coronavirus SARS-CoV-2. Before participating in the study, written consent was signed by all the respondents. A brief instruction letter was provided to the healthcare workers to inform them about the purpose of the research. Due to the seriousness of the pandemic situation and an emergency health response system, a random sample procedure was used to directly reach the healthcare personnel.

A total of 680 FHWs working in 38 hospitals participated in the study. Table 1 shows the demographic characteristics of the sample studied in the current study. The vast majority of the healthcare workers were those who have already been inoculated or had received at least one dose of COVID-19 vaccines. Out of the total participants, 607 (89.26%) were fully vaccinated, whereas, 73 (10.74%) were partially vaccinated. The healthcare workers from both government and private sectors were considered as the target population. As to gender distribution of the collected survey data, 412 (60.58%) were male, whereas, 268 (39.42%) were female healthcare workers. With regards to the age distribution of healthcare workers, 118 (17.35%) were under the age of 25, while 243 (35.73%) aged between 25 and 35 years, whereas, 206 (30.29%), and 113 (16.63%) were between the age of 36–45, and 46–55 years, respectively. Among the total number of healthcare workers, 231 (33.97%) were those who have been infected by SARS-CoV-2 before taking any type of COVID-19 vaccination.

**Table 1:** Characteristics of participants (*n* = 680).

Demographics		Statistics	
	Specifications	Number	%
Gender	Male	412	60.58
	Female	268	39.42
Age	Under-25	118	17.35
	25–35	243	35.73
	36–45	206	30.29
	45–55	113	16.63
Infected with COVID-19	Yes	231	33.97
	No	449	66.02
Family infected with COVID-19	Yes	148	21.76
	No	532	78.23
Number of vaccinated FHWs	Fully	607	89.26
	Partially	73	10.74
Proportion of FHWs	Doctors	231	33.97
	Health technicians	201	29.55
	Nurses	127	18.58
	Midwives	63	9.37
	Community health workers	58	8.53

### Measures

The questionnaire presented measures of all the constructs used in current research including the TPB were those adopted by previous researches in the context of vaccine acceptance including influenza and HPV vaccination (e.g., Kok et al., 2011; Corace et al., 2016; Gallone et al., 2017; Squeri et al., 2017; Alhalaseh et al., 2020). All the constructs of the study presented in Figure 1 were considered latent variables. There are two components to the questionnaire. The first part of the questionnaire includes the demographic information of the healthcare workers, the second part measures the actual behavior of FHWs to uptake COVID-19 vaccines available in the country. The four items taken from the studies of Kok et al. (2011) and Ng et al. (2020) measured the attitude of healthcare workers, whereas, SN was measured through five items adopted from Tickner et al. (2010) and Shmueli (2021). The PBC was measured using three items derived from Li and Li (2020) and Shmueli (2021). To measure the anticipated regret, four items were adopted from the studies conducted by Penţa et al. (2020) and Wolff (2021). Similarly, six items derived from the seminal work of Alhalaseh et al. (2020), Iacob et al. (2021), and Wijayanti et al. (2021) measured the COVID-19 vaccination behavior of healthcare workers. Finally, perceived susceptibility was also measured with four items adopted from the studies of Shmueli (2021) and Zampetakis and Melas (2021). All of the items in this study were assessed using a seven-point Likert Scale from 1 to 7, ranging from strongly disagree to strongly agree, respectively.

### Common Method Bias

Because all of the data for both the dependent and independent variables (IV) were obtained from the same respondents to contribute to the current study, there is a risk of method bias. To limit the possibility of method bias, we ensure that all of the healthcare workers who took part in the study were appropriately briefed about the aim of the study, allowing them to truly understand the questionnaire (including the technical terminologies). The variance inflation factor (VIF) and tolerance (TOL) tests are two more common procedures used for this purpose. The final findings in Table 2 show that the TOL values are greater than 0.1, whereas the observed VIF values are less than 10, indicating that the data is free of collinearity.

**Table 2:** Collinearity assessment.

IV’s	Tolerance	VIF
AR	0.517	1.916
ATT	0.418	3.486
PBC	0.521	2.459
PS	0.429	2.29
SN	0.498	2.096

IV’s, independent variables; ATT, attitude; SN, subjective norm; PBC, perceived behavioral control; AR, anticipated regret; CVB, COVID-19 vaccination behavior; PS, perceived susceptibility; and VIF, variance inflation factor. Source: Estimated results based on the Collinearity assessment by Latan and Noonan (2017).

### Data Analysis

The hypothesized link among the variables was turned into structural equation modeling (SEM), which consists of an outer and an inner model, for further evaluation of the current study model. Smart-PLS 3.3 was used to apply the partial least square structural equation modeling (PLS-SEM) approach. The current research opted for PLS-SEM, a wide range multivariate technique used to statistically examine the relatively complex models and the multivariate relationships among them. It can analyze complex models with a large number of latent variables even with a single item (Shmueli et al., 2019). In the fields of economics and management, the PLS-SEM has shown to be a helpful multivariate analytical approach. Many strategic management experts have acknowledged its flexibility and appropriateness in analyzing numerous interactions among variables (Shmueli et al., 2019).

## Results

This study took a two-step strategy evaluating the suggested research paradigm. The validity and reliability of the scale employed in the study were first verified by assessing the outer mode (measurement model). The inner model (structural model) evaluation was carried out next to test the model fitness as well as recommended relationships between variables. For the overall model evaluation, PLS-SEM version 3.3 was employed.

### Assessment of Measurement Model

Initially, for assessment of the measurement model, this research considered the evaluation of convergent validity and discriminant validity. The specific results of the inner model assessment are depicted in Tables 3, 4. According to Hair et al. (2013), convergent validity refers to how closely the items which measure the same constructs are related to each other. The convergent validity was evaluated *via* factor loadings, composite reliability (CR), and average variance extracted (AVE). Each item’s factor loading ranged from 0.613 to 0.881, above the 0.7 cutoff value. The CR scores ranged from 0.814 to 0.932, indicating a high level of internal consistency (greater than 0.7). Finally, the item loading’s AVE was investigated. The AVE values were between 0.562 and 0.693, over the threshold value of 0.5, indicating that the items explain a significant amount of variation (greater than 50%) in the constructs.

**Table 3:** Assessment of convergent validity (*n* = 680).

Constructs and items	Items	CL	CR	AVE
**Attitude**				
Taking COVID-19 vaccine is a reasonable action for me.	ATT1	0.83	0.89	0.68
I feel safer after being vaccinated against SARS-CoV-2.	ATT2	0.82		
In my opinion, COVID-19 vaccines are effective.	ATT3	0.86		
I feel vaccines are protecting me from SARS-CoV-2.	ATT4	0.73		
**Subjective norm**				
My colleagues forced me to take COVID-19 vaccination.	SN1	0.66	0.85	0.56
My colleagues and family also took COVID-19 vaccines.	SN2	0.82		
COVID-19 vaccination allowed me to protect my patients.	SN3	0.82		
The government pressurized me to get vaccination against the SARS-CoV-2.	SN4	0.61		
I am not allowed to treat patients/work without COVID-19 vaccination card.	SN5	0.73		
**Perceived behavioral control**				
I have enough control to get COVID-19 vaccines.	PBC1	0.79	0.81	0.61
COVID-19 vaccines are easily available for FHCs.	PBC2	0.73		
I can get COVID19 vaccines in every center for vaccination.	PBC3	0.82		
**Anticipated regret**				
I anticipated regret if I did not get vaccination and later contract the SARS-CoV-2.	AR1	0.85	0.88	0.66
I anticipate worry if my friends/family did not get vaccination and later develop serious illness and hospitalization from virus.	AR2	0.84		
I feel less regret of being vaccinated than not vaccinated against SARS-CoV-2.	AR3	0.79		
After vaccination, I anticipate no worry that I can infect others.	AR4	0.75		
**Perceived susceptibility**				
Being a FHW, I considered myself at higher risk of getting infected with SARS-CoV-2.	PS1	0.82	0.91	0.63
I perceived myself more susceptible to experience serious illness and hospitalization if I do not get COVID-19 vaccines.	PS2	0.79		
Being a FHW, I feel my friends and family are at higher risk of getting infected.	PS3	0.81		
Being a FHW, I perceive myself as a source of infection for my patients, friends, and family.	PS4	0.75		
**COVID-19 vaccination behavior**				
I preferred to vaccinate against SARS-CoV-2.	CVB1	0.84	0.93	0.69
I was the earliest beneficiary of COVID-19 vaccine.	CVB2	0.88		
I am ready to take booster shots as well.	CVB3	0.80		
I am ready to take COVID-19 vaccines even if I have to pay for it.	CVB4	0.79		
I recommend COVID-19 vaccines to those who seek my advice.	CVB5	0.81		

CL, cross loadings; CR, composite reliability; and AVE, average variance extracted. ATT, attitude; SN, subjective norm; PBC, perceived behavioral control; AR, anticipated regret; CVB, COVID-19 vaccination behavior; and PS, perceived susceptibility.

**Table 4:** Assessment of discriminant validity (*n* = 680).

Heterotrait–Monotrait ratio (HTMT)					
Anticipated regret					
Attitude	0.765				
COVID-19 vaccination behavior	0.779	0.837			
Perceived behavioral control	0.710	0.795	0.697		
Perceived susceptibility	0.767	0.740	0.804	0.660	
Subjective norm	0.731	0.812	0.764	0.660	0.735

Source: Estimated results based on Henseler et al. (2015) heterotrait–monotrait (HTMT) criterion.

The Henseler heterotrait–monotrait (HTMT) criteria were used to assess discriminant validity. The findings of HTMT are shown in Table 4. The acquired values of HTMT ratios were lower than 0.85, which demonstrates good results for each of the constructs used in the proposed model, according to Henseler et al. (2015) criteria for discriminant validity testing.

### Assessment of Structural Model

**Figure 2:**
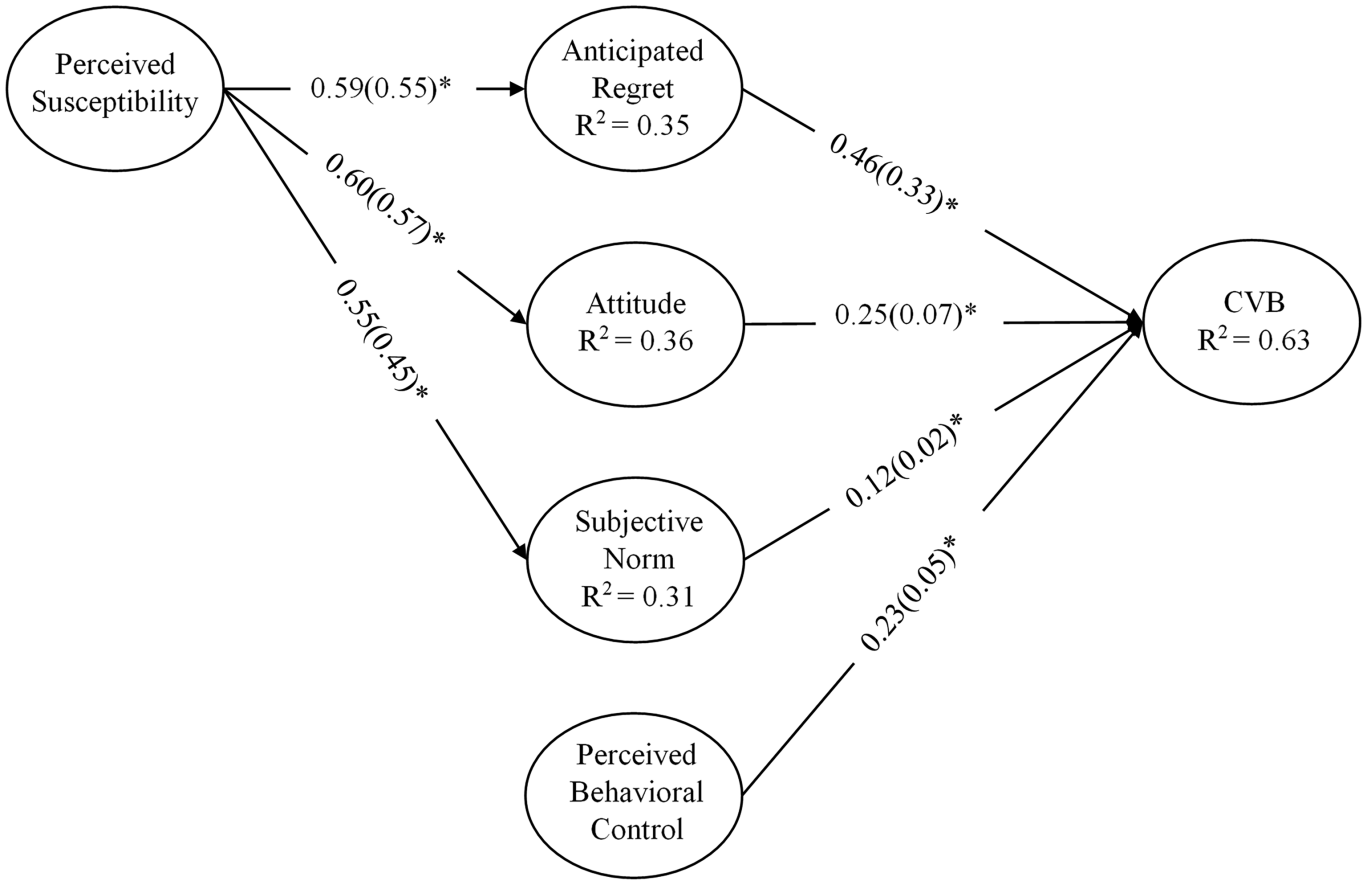
Structural equation modeling (SEM) results of complete data (*n* = 680), CVB: COVID-19 vaccination behavior. The * indicates *p*-values less than 0.01. The figure presents the effect sizes (ƒ^2^) in the parentheses next to each path coefficient (*β*).

The values and dimensions of standardized path coefficients, as well as essential *t*-statistics, including the measurement of *R*^2^ (coefficient of determination), were taken into account for the structural model assessment presented in this study (Figure 2). To measure path coefficients and their relative importance, the researchers used the bootstrapping approach (a resampling technique) to create 5,000 resamples. In addition, the assessment of effect sizes (*f*^2^) for each structural route was evaluated, as proposed by Hair et al. (2017). The research also took Stone-Geisser’s Q^2^ into account while assessing the model’s predictive performance.

Table 5 illustrates the results of β-coefficients, significance values, and effect sizes *f*^2^ for each structural path obtained from the bootstrapping procedure. All of the hypothesized relationships were discovered to be significant. The effect of attitude (ATT→CVB, *β* = 0.258, *t* = 6.512, and *p* ≤ 0.01) on FHWs’ vaccination behavior against SARS-CoV-2 was found to be significant. Subjective norm (SN→CVB, *β* = 0.129, *t* = 3.741, and *p* ≤ 0.01), as well as PBC (PBC→CVB, *β* = 0.231, *t* = 3.230, and *p* ≤ 0.01), were both positively associated with FHWs’ COVID-19 vaccination behavior. As a consequence, the given data supported H1, H2, and H3. Anticipated regret (AR→CVB, *β* = 0.469, *t* = 13.310, and *p* ≤ 0.01) had a stronger effect on FHWs’ COVID-19 vaccine uptake behavior than the TPB components, which sustained H4. With respect to the background factors, perceived susceptibility had a positive influence on AR (PS→AR, *β* = 0.597, *t* = 22.546, and *p* ≤ 0.01), ATT (PS→ATT, *β* = 0.605, *t* = 24.301, and *p* ≤ 0.01), and perceive pressure (PS→SN, *β* = 0.559, *t* = 20.924, and *p* ≤ 0.01), which sustained H5a, H5b, and H5c.

**Table 5:** Structural paths assessment (Hypothesis testing).

Structural paths	*β*-value	*t*-value	*ƒ* ^2^	LL	UL	Results
ATT → CVB	0.258	6.512	0.075	0.184	0.331	Supporting H1
SN → CVB	0.129	3.741	0.027	0.061	0.197	Supporting H2
PBC → CVB	0.231	3.230	0.057	0.041	0.174	Supporting H3
AR → CVB	0.469	13.310	0.337	0.404	0.537	Supporting H4
PS → AR	0.597	22.546	0.555	0.541	0.644	Supporting H5a
PS → ATT	0.605	24.301	0.579	0.554	0.653	Supporting H5b
PS → SN	0.559	20.924	0.455	0.510	0.614	Supporting H5c

In current study, the vaccination behavior of frontline health workers against Coronavirus disease 2019 pandemic in Pakistan was measured. ATT, attitude; SN, subjective norm; PBC, perceived behavioral control; CVB, COVID-19 vaccination behavior; AR, anticipated regret; and PS, perceived susceptibility. LL, lower limit, UL, upper limit at 99 percent CI.

To evaluate the measurement of effect sizes (*f*^2^), the Cohen criteria of small, medium, and large size effects, that is, 0.02, 0.15, and 0.35, were utilized (Cohen, 1970). The effect sizes (small-to-large) of all the factors surpassed the minimal threshold criteria of 0.02, indicating that they had a significant impact on the dependent variables. The effect of perceived susceptibility on anticipated regret, attitude, and subjective norm to uptake COVID-19 vaccination was quite substantial.

Furthermore, the current research looked at the coefficient of determination (*R*^2^) and predictive relevance (*Q*^2^) of exogenous variables on endogenous variables. The adjusted *R*^2^ value for the endogenous variable (COVID-19 vaccination behavior, CVB) was determined to be 0.635. This means that the exogenous factors in the current study (ATT, AR, SN, and PBC) account for 63.5 percent of the variance in the endogenous variable (CVB). The *R*^2^ values for endogenous variables (AR, ATT, and SN) were determined to be 0.356, 0.366, and 0.312, respectively. Perceived susceptibility explains 35.6, 36.6, and 31.2 percent of differences in expected regret, attitude, and subjective norm, respectively.

We also performed the PLS predict based on the procedures recommended by Shmueli et al. (2019). The cross-validation approach with holdout sampling was used to evaluate the predictive validity of the research model. Table 6 summarizes the overall findings of the assessment. To begin, the *Q*^2^ values (i.e., the difference between the PLS path model and prediction of the simple mean) were calculated, and the results were 0.186, 0.197, 0.324, 0.122 for AR, ATT, CVB, and SN, respectively. The *Q*^2^ findings indicate that the suggested model has a good predictive performance. Second, the linear regression model (LM) was used to create predictions, as recommended by Shmueli et al. (2019). Finally, the findings of the PLS and LM comparison show that the LM outcomes have smaller prediction errors in both root mean square error (RMSE) and mean absolute error (MAE), indicating that the model has strong predictive capacity.

**Table 6:** PLS predict assessment.

PLS prediction summary									
		**Q2**							
AR		0.186							
ATT		0.197							
CVB		0.324							
SN		0.122							
**PLS prediction summary**									
	**PLS**			**LM**			**PLS-LM**		
	**RMSE**	**MAE**	**Q2** **predict**	**RMSE**	**MAE**	**Q2** **predict**	**RMSE**	**MAE**	**Q2** **predict**
AR3	1.657	1.34	0.159	1.651	1.321	0.166	0.006	0.019	−0.007
AR2	1.654	1.321	0.201	1.658	1.323	0.198	−0.004	−0.002	0.003
AR4	1.614	1.293	0.162	1.562	1.242	0.214	0.052	0.051	−0.052
AR1	1.556	1.238	0.222	1.544	1.205	0.234	0.012	0.033	−0.012
Att1	1.777	1.381	0.167	1.547	1.08	0.367	0.23	0.301	−0.2
Att3	1.656	1.306	0.241	1.599	1.232	0.291	0.057	0.074	−0.05
Att4	1.761	1.448	0.194	1.726	1.394	0.225	0.035	0.054	−0.031
Att2	1.698	1.336	0.188	1.591	1.181	0.287	0.107	0.155	−0.099
CVB1	1.605	1.286	0.235	1.587	1.221	0.251	0.018	0.065	−0.016
CVB6	1.579	1.239	0.216	1.564	1.206	0.23	0.015	0.033	−0.014
CVB5	1.616	1.325	0.225	1.604	1.273	0.236	0.012	0.052	−0.011
CVB3	1.587	1.266	0.262	1.555	1.178	0.291	0.032	0.088	−0.029
CVB4	1.569	1.263	0.258	1.553	1.195	0.273	0.016	0.068	−0.015
CVB2	1.651	1.326	0.225	1.648	1.284	0.228	0.003	0.042	−0.003
SN2	1.784	1.488	0.152	1.791	1.485	0.146	−0.007	0.003	0.006
SN5	1.803	1.493	0.09	1.795	1.483	0.099	0.008	0.01	−0.009
SN4	1.907	1.597	0.015	1.882	1.567	0.041	0.025	0.03	−0.026
SN1	1.667	1.313	0.171	1.59	1.214	0.246	0.077	0.099	−0.075
SN3	1.721	1.409	0.176	1.724	1.399	0.173	−0.003	0.01	0.003

ATT, attitude; SN, subjective norm; PBC, perceived behavioral control; AR, anticipated regret; CVB, COVID-19 vaccination behavior; PS, perceived susceptibility; LM, linear regression model; RMSE, root mean square error; and MAE, mean absolute error.

### Mediation Effect

The theoretical model of the current research also proposed the indirect effect of perceived susceptibility on the COVID-19 vaccination uptake behavior through anticipated regret, attitude, and subjective norm (H6a, H6b, and H6c). To assess the mediation impact, the bootstrapping approach was used to create 5,000 resamples, as recommended by Hair et al. (2019). The results obtained from all mediation routes in the proposed model were reported using the Smart-PLS function of specific indirect impact. Table 7 summarizes the mediation effects of overall findings. The indirect impact of perceived susceptibility on COVID-19 vaccination behavior *via* the mediation of AR (PS→AR→CVB, *β* = 0.282, *t* = 10.527, and *p* ≤ 0.01) was significant. Similarly, through the mediation of ATT (PS→ATT→CVB, *β* = 0.156, *t* = 5.952, and *p* ≤ 0.01) and SN (PS→SN→CVB, *β* = 0.072, *t* = 3.53, and *p* ≤ 0.01), the indirect impact of perceived susceptibility on COVID-19 vaccination behavior was shown to be significant. As a result of the current study’s findings, we infer that perceived susceptibility influences COVID-19 vaccination behavior indirectly *via* the mediation of anticipated regret, attitude, and subjective norm.

**Table 7:** Mediation effect.

Structural paths	*β*-value	*t*-value	*p*-values	LL	LL	Status
PS → AR → CVB	0.282	10.527	0.00	0.229	0.333	Supporting H6a
PS → ATT → CVB	0.156	5.952	0.00	0.112	0.208	Supporting H6b
PS → SN → CVB	0.072	3.53	0.00	0.034	0.112	Supporting H6c

Significance at *p* ≤ 0.01. ATT, attitude; SN, subjective norm; PBC, perceived behavioral control; CVB, COVID-19 vaccination behavior; AR, anticipated regret; and PS, perceived susceptibility. LL, lower limit, UL, upper limit at 99% CI.

## Discussion and Conclusion

The ongoing pandemic has triggered new healthcare catastrophes since the number of COVID-19 infected patients has dramatically increased worldwide, the SARS-CoV-2 signs and symptoms were examined in combination with the quick development of a vaccine to combat this lethal disease. The WHO has made global efforts to control the transmission of illness and enhance treatment methods to reduce morbidity and mortality. The steps taken to contain the pandemic bought time for the creation of effective and safe COVID-19 vaccinations. Right now, numerous COVID-19 vaccines have been approved by WHO for emergency uses, while more than a hundred vaccines are being tested in various phases of development (GAVI The Vaccine Alliance, 2021). Recently, the COVID-19 vaccines have reached billions of individuals around the world, and the evidence is mounting that no matter which vaccine you decide to take, they still provide life-saving protection against the deadly virus. However, it is not a vaccine that will halt the pandemic, but vaccination. The excessive efforts for COVID-19 vaccines’ availability cannot promise the end of the pandemic due to the ongoing vaccine hesitancy and anti-vaccine campaigns worldwide. Given the enormous role of inoculated healthcare workers in shaping the general population’s willingness to vaccinate against a disease, the current research thus aims to investigate the actual behavior of FHWs in Pakistan to vaccinate against SARS-CoV-2. To the best of the authors’ knowledge, this is the first study in Pakistan to examine theoretical and psychological factors in determining intended behavior using one of the most important socio-psychological frameworks, the TPB, as well as additional factors, such as anticipated regret and perceived susceptibility. The findings exposed that TPB is a valuable framework for predicting numerous health-related behaviors and has significant explanatory power.

Initially, while investigating the direct relation among classical components of TPB, we found that attitude of FHWs toward COVID-19 vaccination predicts their behavioral intentions, consequently, up taking the vaccines to immunize against COVID-19, supporting H1. The results confirmed that this behavior is strongly supported by a positive attitude toward available vaccines. This result suggests that FHWs have strong beliefs about the perceived benefits and adverse effects of vaccinating and not vaccinating against SARS-CoV-2, respectively. Our findings are in line with those of Li et al. (2021), Verger et al. (2021), and Wang et al. (2021), who reported a favorable attitude among healthcare professionals to get COVID-19 vaccination in China, France, and Belgium, respectively.

Secondly, the perceived pressure from important others also affects the COVID-19 vaccination behavior of healthcare workers in Pakistan, which supports H2. The positive association between SN and the behavior to get COVID-19 vaccines reflect the discussions of the importance of the vaccines with friends and family, which influence their willingness to immunize against SARS-CoV-2. In addition, the pressure from health authorities and NCOC also affect the willingness of FHWs in Pakistan to take COVID-19 vaccination at priority bases. Our findings are in line with those of Wolff (2021), who discovered subjective norm to be an important predictor of COVID-19 vaccination intentions.

Thirdly, our results also provided support for H3, which suggests that the FHWs in Pakistan have significant behavioral control to get the required doses of COVID-19 vaccines. This suggests that the availability of COVID-19 vaccines is not an obstacle for FHWs in Pakistan. The NCOC of Pakistan has approved several COVID vaccines which have been rolled out in different phases. Considering the seriousness of the pandemic situation in Pakistan and the significant role of healthcare workers in the success of the national immunization program against SARS-CoV-2, the FHWs were scheduled to be the first beneficiaries of the vaccination program. At the moment, COVID-19 vaccines are freely available for all citizens (12 years old and above) of Pakistan at their nearest health facility. Our findings are in confirmatory with the recently published work of Harapan et al. (2020) and Qattan et al. (2021).

Finally, H4 received strong support which suggests that the FHWs in Pakistan have less anticipated regret to vaccinate against SARS-CoV-2 than the anticipated regret from not vaccinating. Our results further suggest that anticipated regret is the most prominent predictor of healthcare workers getting COVID-19 vaccines. The results of the present study are consistent with previous research conducted by Brewer et al. (2016), Iacob et al. (2021), and Wolff (2021) about the anticipated regret and administering vaccines. As discussed earlier, this could be attributed to the fact that individuals anticipate less regret for easily admissible decisions than for less admissible ones. Based on the constant exposure to the infected COVID-19 patients, the FHWs in the country are well aware that the disadvantages of not vaccinating themselves are greater than vaccinating.

With regards to the background variable, H5a received support suggesting that the healthcare workers are perceived as being at higher risk to get infected by SARS-CoV-2, affecting their anticipated regret, which further reflects higher rates of COVID-19 vaccination. Brewer et al. (2016) and Penţa et al. (2020) discovered similar considerations in the context of HPV vaccinations. Furthermore, H5b got widespread support since perceived susceptibility was found to be associated with attitude, confirming previous research (Gallone et al., 2017; Squeri et al., 2017; Taebi et al., 2019). For instance, only those medical students in Italy who perceive themselves at higher risk to catch flu have positive attitudes to get influenza vaccines (Gallone et al., 2017). Similarly, the Iranian population who perceive themselves as at higher risk to contract HPV believes that getting HPV vaccination is important for them (Taebi et al., 2019). In addition, Squeri et al. (2017) explored the healthcare workers with a higher perceived susceptibility to getting infected with a specific disease have positive evaluation toward vaccination. Moreover, our results highlighted that perceived susceptibility has an association with SN to uptake COVID-19 vaccines, resultantly supporting H5c. The positive effect of perceived susceptibility on SN articulates higher concerns regarding COVID-19 vaccination. Considering the seriousness of the ongoing pandemic, the Pakistani FHWs believe themselves at higher risk of contracting the virus, therefore they are more inclined to follow the opinions of important referents and instructions from health authorities. Our findings support findings of Li and Li (2020) that interpersonal interaction with key others about the danger connected with HPV vaccination positively improves the SN’s willingness to adopt the HPV vaccine.

Finally, we checked whether anticipated regret, attitude, and subjective norm lead to the mediation between perceived susceptibility and COVID-19 vaccination behavior of FHWs in Pakistan. The results revealed that perceived susceptibility is associated with the COVID-19 vaccination behavior of Pakistani FHWs through its substantial effect on anticipated regret (H6a), attitude (H6b), and subjective norm (H6c), which accepted the overall mediation hypothesis. The results further suggest that the FHWs as being more susceptible to SARS-CoV-2 are taking COVID-19 vaccination because they have lower anticipated regret, having favorable evaluation of vaccines, and thus listing to the suggestions from their important referents.

The proposed vaccination model, which used the standard TPB framework to incorporate anticipated regret and perceived susceptibility, produced reliable findings in explaining the COVID-19 vaccination behavior of FHWs in Pakistan. However, the additional variables were added to the classical TPB model to assess their role in shaping the behavior of FHWs to uptake COVDI-19 vaccination, which can be considered as an operationalization within the original framework, and has never been applied nor validated by previous researches. We believe that our study is novel in comparison to the existing COVID-19 vaccines’ literature for three reasons. Firstly, in our study, a self-reported actual vaccination behavior is assessed, while the existing research on COVID-19 vaccination is limited to the intention phase only. Secondly, the data was collected through well-organized self-administered questionnaires with a brief instructions letter physically distributed among FHWs which increased the predictive power of the hypothesized model. Thirdly, in the case of COVID-19 vaccination, the role of anticipated regret and perceived susceptibility was questioned, given its heuristic role within the cognitive process existing behind behavioral execution.

Regardless of its strength, the current research also has a few caveats. Firstly, due to Pakistan’s epidemic control policies, lockdowns, and restrictions on interprovincial movement, in particular, the data was collected in one province, thus the generalizability of the results to entire healthcare workers in the country is questionable. Secondly, the data was collected from only those healthcare workers who have been fully vaccinated or have received at least one shot of the vaccine having a vaccination certificate from the national database and registration authority (NADRA) Pakistan. Thus, the generalization of willingness to uptake COVID vaccines among entire healthcare workers in Pakistan is also questionable. Thirdly, some additional factors like perceived severity, cost of vaccination, and perceived threat can also be considered to accompany the proposed relationships in current research. Lastly, the current research investigated the vaccination behavior of healthcare workers regardless of the type, safety, and efficacy of the vaccine they have inoculated, which can be investigated by future research.

## Implications and Future Research Directions

The current study’s findings contribute to the limited literature on COVID-19 vaccination behavior in a variety of ways. To begin with, it has added to the little study on vaccination behavior among Pakistan’s frontline HCWs in terms of expected regret and perceived susceptibility during the pandemic. Second, the TPB was used to explore the antecedents of FHWs choice to uptake COVID-19 immunization in order to combat the virus’ spread and prevent Pakistan’s healthcare system from collapsing. Third, the association between perceived susceptibility and COVID-19 vaccination behavior was presented through the mediation of anticipated regret, attitude, and subjective norm, which has never been tested nor applied in previous researches.

The current findings of our research have several implications as well. The results confirmed the importance of perceived risk and anticipated regret in taking COVID-19 vaccination among healthcare workers. The results of current research should be used as a potential source of inspiration to reduce hesitancy and build vaccination confidence among the general population. Based on their own experience, these FHWc should educate their family, friends, patients, as well as the general population regarding the benefits of COVID vaccines and the potential negative health consequences of illness they can experience. Secondly, the health communication agents including health authorities, social media, and non-profit organizations should endorse/consider these healthcare workers as ambassadors to communicate the vaccination benefits, apply more positive social norms, and build confidence among the general population in taking COVID-19 vaccination to get herd immunity. The FHWs’ recommendations that are widely visible for the general population can effectively promote the COVID-19 vaccination process.

## Data Availability Statement

The raw data supporting the conclusions of this article will be made available by the authors, without undue reservation.

## Ethics Statement

The studies involving human participants were reviewed and approved by the ethics committee at China University of Geosciences, Wuhan, China. The patients/participants provided their written informed consent to participate in this study.

## Author Contributions

MK and SC conceived and designed the study. MK, RA, and HA collected the data. MK and SC developed the theoretical framework. MK and NK performed the data analysis. SC, AN, and JA verified the analytical methods. MK wrote the first draft. SC, MI, and MAS substantially revised the manuscript. All authors discussed the results and contributed to the final manuscript.

## Conflict of Interest

The authors declare that the research was conducted in the absence of any commercial or financial relationships that could be construed as a potential conflict of interest.

## Publisher’s Note

All claims expressed in this article are solely those of the authors and do not necessarily represent those of their affiliated organizations, or those of the publisher, the editors and the reviewers. Any product that may be evaluated in this article, or claim that may be made by its manufacturer, is not guaranteed or endorsed by the publisher.
